# AGMSF-VMUNet: attention-guided multi-scale feature fusion VM-UNet for crop pest detection

**DOI:** 10.3389/fpls.2026.1855769

**Published:** 2026-07-13

**Authors:** Shanwen Zhang, Xuqi Wang, Cong Xu

**Affiliations:** School of Electronic Information, XiJing University, Xi’an, China

**Keywords:** AGMSF-VMUNet, attention gate skip connections, crop pest detection, multi-scale feature fusion, state space model

## Abstract

Crop pest detection (CPD) in field environments is critical yet challenging due to high pest variability, irregular shapes, and complex backgrounds. Conventional CNNs suffer from limited receptive fields, while Vision Transformers (ViTs) are hindered by quadratic computational complexity, limiting their practical deployment. To address these issues, an attention-guided multi-scale feature fusion VM-UNet (AGMSF-VMUNet) model for CPD is constructed. The model integrates three key components: Multi-Scale VSS (MSVSS) blocks that combine state-space models with multi-scale dilated convolutions to capture both global context and local details; Attention Gate Skip Connections (AGSC) that dynamically recalibrate encoder features to enhance pest-relevant information while suppressing background clutter; and a progressive Multi-scale Feature Fusion (MSFF) module that adaptively integrates features across decoder stages through spatial attention. Extensive experiments on the IP102 dataset demonstrate that AGMSF-VMUNet achieves state-of-the-art segmentation performance with 77.22% mean Intersection over Union (mIoU) and 80.21% Dice Similarity Coefficient (DSC). Ablation studies confirm the synergistic contribution of each component. This work provides an effective solution for intelligent crop pest monitoring in real-world agricultural applications.

## 1 Introduction

Crop pest detection (CPD) in field environments is a critical yet challenging task for precision agriculture, enabling timely intervention to minimize crop losses and reduce pesticide overuse (Abdullah et al., 2023; Kiobia et al., 2023). Accurate CPD directly supports sustainable agricultural practices by facilitating targeted pest management. However, field conditions present substantial challenges: pests exhibit diverse shapes, variable postures, and small sizes; environmental factors such as changing lighting, occlusion, and complex backgrounds further complicate detection; and intra-species morphological variations across different life cycles increase classification difficulty (Zhang et al., 2024; Nyawose et al., 2025). These factors necessitate advanced computational approaches capable of extracting both global context and local discriminative features.

Traditional pest detection relies on manual inspection, which is time-consuming and inefficient (Domingues et al., 2022; Zhang et al., 2025). Early machine learning approaches partially automated this process but suffer from fundamental limitations, including heavy reliance on manual feature engineering and poor generalization to field variations (Pattnaik and Parvathi, 2022; Reddy and Ramesh, 2024). These limitations have driven the shift toward deep learning for more robust CPD solutions.

Deep learning, particularly convolutional neural networks (CNNs) such as YOLO and U-Net, has revolutionized CPD by enabling automatic feature extraction (Shoaib et al., 2023; Biradar and Hosalli, 2024). While CNNs excel at capturing local features, they struggle to model long-range dependencies due to their limited receptive fields (Xu et al., 2022). Vision Transformers (ViTs) address this issue through self-attention mechanisms but introduce quadratic computational complexity, hindering their efficiency in high-resolution and real-time applications (Wang et al., 2024; Zhang et al., 2025).

Recent advances in state space models (SSMs), particularly Mamba, offer a compelling alternative by modeling long-range dependencies with linear computational complexity (Xiang et al., 2025). Visual Mamba (VMamba) and its variants (e.g., VM-UNet) have shown promise in various vision tasks by combining SSMs with U-Net architectures (Saranya et al., 2024; Mayya and Alkayem, 2025). Despite these advances, existing CPD methods still face three critical challenges when deployed in real-world agricultural settings: (1) Loss of fine-grained details during downsampling, (2) Complex background interference that obscures pest features, and (3) Multi-scale variation from tiny aphids to large caterpillars.

To address these challenges, Attention-Guided Multi-Scale Feature Fusion VM-UNet(AGMSF-VMUNet) for accurate CPD is proposed. The main contributions are summarized as follows:

1. AGMSF-VMUNet combines VM-UNet, multi-scale feature extraction, and attention mechanisms to balance fine-grained detail preservation with deep semantic understanding.
2. Multi-Scale VSS (MSVSS) and Attention Gate Skip Connection (AGSC) capture both local details and global context with linear complexity while dynamically filtering encoder features to enhance pest-relevant regions and suppress background interference.
3. Multi-scale Feature Fusion (MSFF) adaptively integrates features across decoder stages through spatial attention, ensuring effective preservation of fine-grained pest features throughout the network.

The remainder of this paper is organized as follows. Section 2 reviews related work on CPD. Section 3 details the AGMSF-VMUNet architecture and its components. Sections 4 and 5 present experimental results and analysis. Section 6 concludes the paper with future research directions.

## 2 Related work

Based on underlying architectures, existing CPD methods can be categorized into four classes: machine learning (ML), convolutional neural networks (CNNs), Vision Transformers (ViTs), state-space models (SSMs), and related advances. This section reviews representative works in each category, analyzing their advantages and limitations.

### 2.1 Machine learning methods

Early CPD methods relied on traditional machine learning approaches. These methods typically involve handcrafted feature extraction (e.g., texture, color, shape descriptors) followed by classifier training using algorithms such as Support Vector Machines, Random Forests, or Decision Trees (Pattnaik and Parvathi, 2022; Reddy and Ramesh, 2024). Despite their interpretability and low computational requirements, ML methods suffer from four fundamental limitations that hinder practical deployment: (1) heavy dependence on manual feature engineering, making adaptive mining of deep discriminative information difficult; (2) poor generalization to field variations in lighting, background, and pest posture; (3) lack of end-to-end learning capabilities, often requiring multi-stage pipelines that complicate optimization; and (4) scalability bottlenecks when processing large-scale field image data (Domingues et al., 2022; Zhang et al., 2025). These limitations have fundamentally driven the shift toward deep learning for more robust and accurate CPD solutions.

### 2.2 Convolutional neural network methods

Deep learning, particularly CNNs, has become dominant in CPD by enabling automatic feature extraction for image classification, object detection, and segmentation tasks (Shoaib et al., 2023). YOLO variants have significantly improved small-target detection and real-time performance through advances like multi-scale fusion, attention mechanisms, and lightweight design (Xu et al., 2022; Biradar and Hosalli, 2024). U-Net and its derivatives have demonstrated strong potential in agricultural image segmentation due to their encoder-decoder structure and skip connections (Wang et al., 2024; Zhang et al., 2025). For instance, Biradar and Hosalli (Shoaib et al., 2023) proposed a novel U-Net with hybrid deep learning mechanisms for crop pest segmentation. Wang et al (Zhang et al., 2025). developed a dilated multi-scale attention U-Net for insect pest detection, achieving improved performance through multi-scale receptive fields. Xiang et al (Wang et al., 2024). enhanced YOLOv7 with U-Net components, combining the strengths of both architectures.

Despite their strong local feature extraction capabilities, CNN-based methods inherently struggle with capturing long-range dependencies due to their limited receptive fields (Mayya and Alkayem, 2025; Xiang et al., 2025). This limitation is particularly problematic for CPD, where contextual information from surrounding regions is crucial for distinguishing pests from visually similar backgrounds. Furthermore, the fixed receptive field of CNNs makes it difficult to simultaneously capture fine-grained details (e.g., antennae, legs) and global structural information.

### 2.3 Vision transformer methods

Vision Transformers (ViTs) overcome CNN limitations by leveraging self-attention mechanisms to capture global context, enabling effective modeling of long-range dependencies (Saranya et al., 2024). For CPD applications, Liu et al. (2025) proposed an end-to-end pest segmentation method using transformer feature compensation and regional attention, recovering downsampling loss without additional computational overhead. Cao et al. (2023) developed SwinU-Net, combining U-Net with Swin Transformer for improved medical image segmentation, which has been adapted for agricultural applications.

Despite their global modeling advantages, Transformers and ViTs are limited by quadratic computational complexity, and pure ViTs lack the inherent spatial priors of CNNs. Recently, lightweight multi-scale architectures have emerged as a promising direction for agricultural pest detection, particularly for edge deployment scenarios. Yang et al (Yang et al., 2025). proposed lightweight networks (AgriLiteNet) for Agriculture. It is an efficient lightweight neural network integrating MobileNetV3 for local feature extraction and a streamlined Swin Transformer for global modeling.

### 2.4 State space model methods

Recent advances in state space models (SSMs), particularly Mamba, offer a compelling alternative by modeling long-range dependencies with linear computational complexity (Rahman et al., 2024). The Mamba architecture is renowned for its ability to handle long sequences and global contextual information efficiently, making it well-suited for high-resolution agricultural imagery. Visual Mamba (VMamba) integrates the Visual State Space (VSS) with attention mechanisms, achieving strong results in video understanding and remote sensing tasks (Wang et al., 2024). Mamba-UNet (Ruan et al., 2024) combines U-Net with Mamba, while VM-UNet (Liu et al., 2024) adopts a VMamba-based encoder-decoder with skip connections to preserve multi-scale spatial information. Several variants have emerged: Swin-UMamba (Liao et al., 2024) introduces ImageNet-based pretraining; LightM-UNet (Yang et al., 2025) focuses on lightweight design; MSV-Mamba (Wang et al., 2024) combines large-window Mamba with hierarchical feature fusion; and MedMamba (Zhang et al., 2023) integrates convolutional local features with SSM long-range capabilities for medical image classification. For CPD specifically, InsectMamba (Wu et al., 2019) integrates SSM, CNN, multi-scale attention, and MLP, achieving high accuracy across datasets despite data annotation requirements. However, InsectMamba was designed for classification rather than segmentation, limiting its applicability for precise pest localization.

### 2.5 Related advances

Several recent advances inform our approach. Multi-modal attention fusion (Yuan et al., 2025) integrates complementary information in remote sensing, aligning with our AGSC and MSFF modules. Pseudo-labeling strategies (Zhang et al., 2025) address limited labeled data in medical imaging, a challenge also faced in pest detection. Progressive sample selection with contrastive loss (Jin et al., 2026) handles noisy labels, relevant to IP102’s class imbalance. These innovations inform our attention-guided feature fusion and robust training strategies.

The complementary advantages of the existing methods and the specific challenges faced by CPD, including the loss of fine details during downsampling, complex background interference, and multi-scale pest variations, AGMSF-VMUNet for CPD is proposed. It is an attention-guided multi-scale feature fusion VM-UNet model.

## 3 Attention-guided multi-scale feature fusion VM-UNet

**Figure 1:**
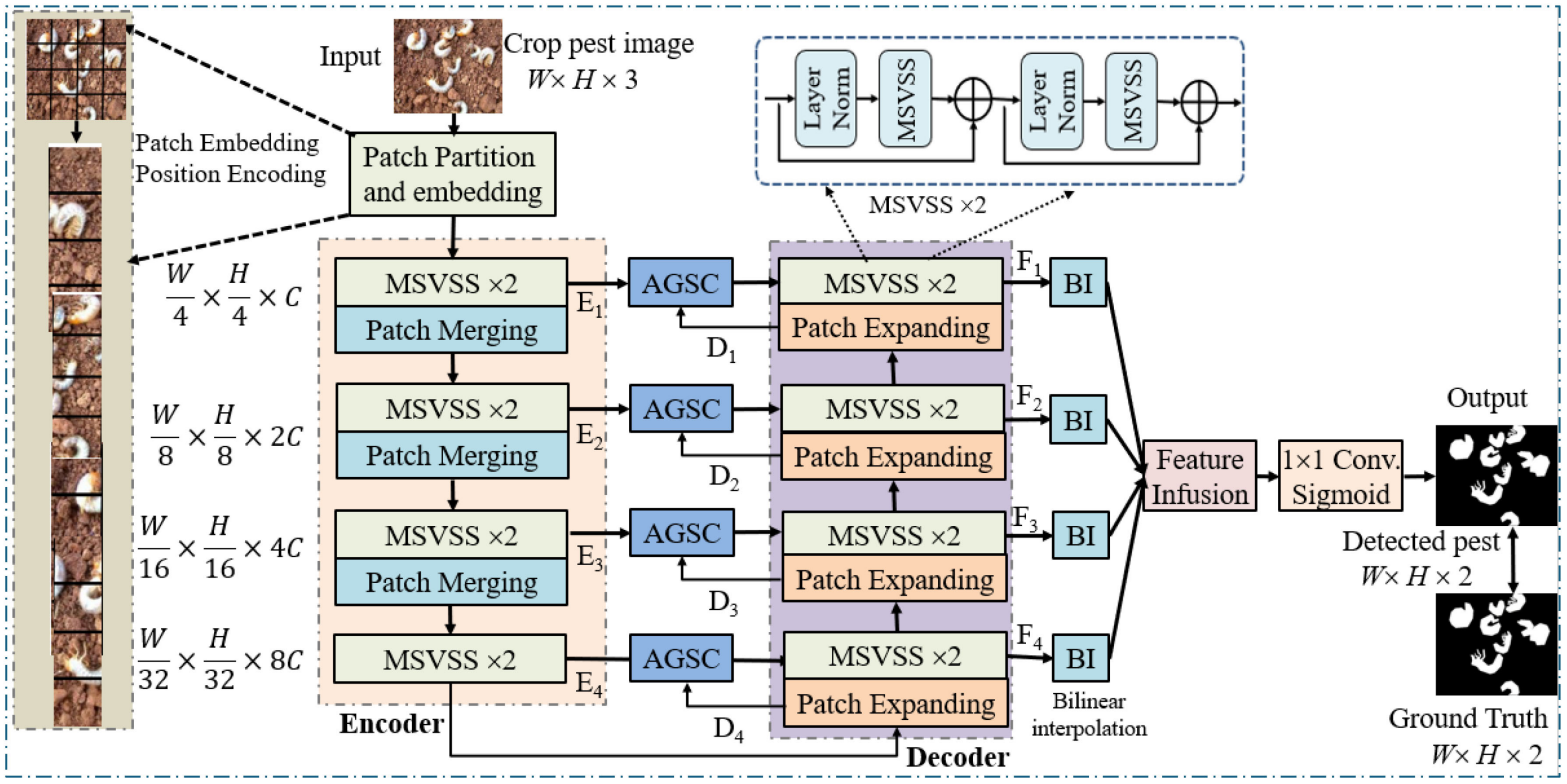
The architecture of AGMSF-VMUNet.

**Figure 2:**
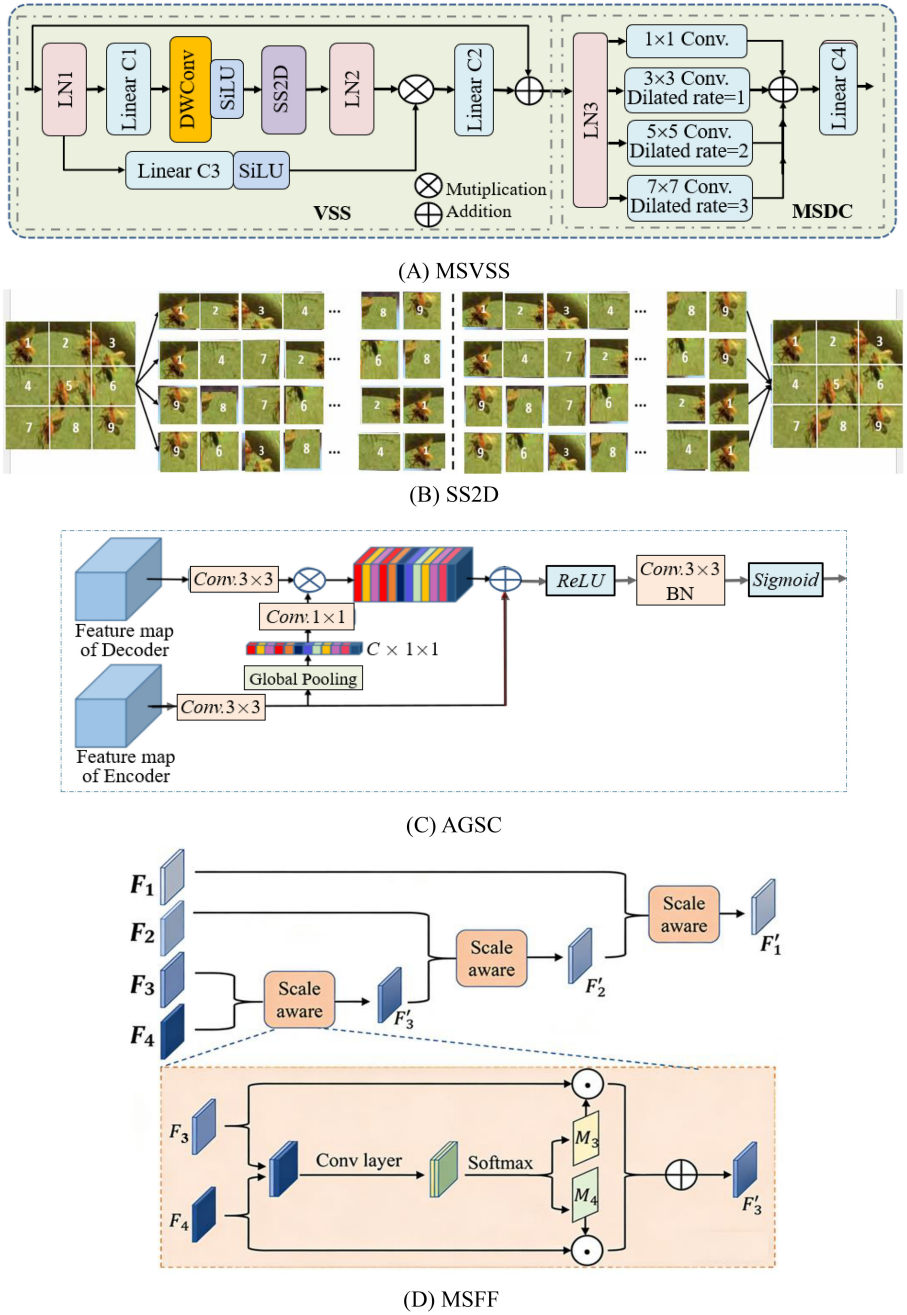
The structures of the main components of AGMSF-VMUNet. **(A)** MSVSS. **(B)** SS2D. **(C)** AGSC. **(D)** MSFF.

**Figure 1** illustrates the architecture of the proposed Attention-Guided Multi-Scale Feature Fusion VM-UNet (AGMSF-VMUNet). The structures of its main components are shown in **Figure 2**.

### 3.1 Overview of AGMSF-VMUNet

AGMSF-UNet adopts a symmetric encoder-decoder architecture with VM-UNet as the backbone. The model consists of four main components: an encoder, a decoder, four Attention Gate Skip Connections (AGSC), and a progressive Multi-scale Feature Fusion module (MSFF).

MSVSS blocks. This block serves as the fundamental building unit in both the encoder and decoder. The encoder begins with a patch embedding layer followed by four hierarchical stages. The input image of size H×W×3 is first divided into non-overlapping 4×4 patches and linearly projected to a C-dimensional embedding space (default C = 96). Each encoding stage employs MSVSS blocks for feature extraction. Between consecutive stages, patch merging operations are performed to gradually reduce spatial resolution while increasing channel dimension, forming a typical pyramid structure: H/4×W/4×C → H/8×W/8×2C → H/16×W/16×4C → H/32×W/32×8C.

Symmetrically, the decoder restores spatial resolution through patch expanding layers and MSVSS blocks, generating feature maps at progressively increasing resolutions: H/32×W/32×8C → H/16×W/16×4C → H/8×W/8×2C → H/4×W/4×C.

Attention Gate Skip Connections (AGSC). Features from corresponding encoder and decoder stages are integrated via AGSC. These attention gates selectively filter encoder features before fusion, suppressing irrelevant background information while enhancing pest-relevant regions, thereby improving segmentation accuracy.

Multi-scale Feature Fusion module (MSFF). Embedded across the decoding stages, the MSFF is a core innovation of this model. It achieves progressive refinement of multi-scale features through spatial attention mechanisms operating between adjacent scales, significantly enhancing the representational capacity of discriminative pest features.

Comparison with VM-UNet. Compared with the traditional VM-UNet architecture, AGMSF-UNet introduces MSVSS blocks in both encoder and decoder to capture global dependencies and multi-scale convolutional features, whereas the standard VM-UNet relies solely on convolutional layers for local feature extraction. Furthermore, the integration of the MSFF in skip connections and decoding stages distinguishes AGMSF-UNet from VM-UNet, enabling adaptive MSFF and overcoming the limitation of simple skip connections that inadequately transmit fine details. This design combines the efficient global modeling capability of state-space models with the feature selectivity of attention mechanisms, forming a powerful system that simultaneously captures global context and local details—making it particularly suitable for precise segmentation of tiny pest features in complex field environments.

### 3.2 MSVSS

As shown in **Figure 2A**, the Multi-Scale VSS (MSVSS) block consists of a VSS module and a Multi-Scale Dilated Convolution (MSDC) module (Zhang et al., 2023). It extends the standard VSS block by incorporating multi-scale convolutional features and channel attention mechanisms to enhance representation learning.

VSS Module. Following Layer Normalization (LN1), the input feature map is split into three parallel branches. The first branch consists of a linear layer (C1), depthwise convolution (DWConv), SiLU activation, SS2D, and Layer Normalization (LN2). The second branch comprises a linear layer (C2) and SiLU activation. The third branch is a residual connection. The linear layers preserve the original feature representation. SS2D performs selective scanning along four directions (horizontal, vertical, and their reverse paths) to capture long-range dependencies with linear computational complexity. The SS2D output is then element-wise multiplied with the second branch features shown in **Figure 2B**, enabling selective fusion of global context with local details. Finally, the fused features pass through a linear layer and are combined with the residual connection from the module input to produce the MSVSS output.

MSDC is a modified Inception module consisting of four parallel convolutional paths with different receptive fields: a 1×1 convolution for point-wise feature transformation, a 3×3 convolution with dilation rate 1 for local context, a 5×5 convolution with dilation rate 3 for an expanded receptive field, and a 7×7 convolution with dilation rate 3 for further expanded receptive field. The multi-scale outputs are fused and processed by a Layer Normalization block to adaptively recalibrate channel-wise feature responses. A final linear layer projects the refined features. The process of MSVSS is as show in Equation 1:

(1) $$\begin{array}{l} F_{out}^{MSVSS}=Lin4(MSDC(LN3(VSS(V_{in}))))\\ VSS(V_{in})=Lin2(LN2(SS2D(SiLU(DWConv((Lin1(LN1(V_{in}))))))⊗V_{ls})+V_{in}\\ V_{ls}=SiLU(Lin3(LN1(V_{in})))\\ MSDC(x)=(Conv_{1\times 1}(x)⊕Conv_{3\times 3}(x)⊕Conv_{5\times 5}(x)⊕Conv_{7\times 7}(x)) \end{array}$$

where $V_{in},F_{out}^{MSVSS}$ are the input and output feature maps of MSVSS, *LN*(·), *Lin*(·), *SiLU*(·), *SS*2*D*(·), *DWConv*(·) are layer normalization, linear projecting, SiLU activation, SS2D and DWConv operations, respectively, VSS(.) is the VSS operation, and *MSDC*(·) is MSDC operation.

By integrating multi-scale convolutions, channel attention, and selective scanning, MSVSS effectively captures both fine-grained local details and global contextual information essential for accurate CPD under complex field conditions.

### 3.3 AGSC

To enhance feature transmission between encoder and decoder while suppressing irrelevant background information, Attention Gate Skip Connections (AGSC) are introduced into AGMSF-UNet as shown in **Figure 2C**. AGSC dynamically recalibrates encoder features before they are fused with decoder features, ensuring that pest-relevant information is prioritized during the upsampling process.

Given feature maps *M*_1_ from the encoder and *M*_2_ from the decoder, *M*_1_ and *M*_2_ first applies a 3×3 convolution to capture the corresponding local features $M_{1}^{\prime }$ and $M_{2}^{\prime }$. The convolved features $M_{1}^{\prime }$ are then passed through a global pooling operation, which aggregates spatial information into a channel descriptor of dimensions *C*×1×1, then is multiplied with $M_{2}^{\prime }$, subsequently processed by two fully connected (or convolutional) layers with ReLU activation, 3×3 convolution, followed by a Sigmoid function to generate channel-wise attention weights. The Sigmoid output is then normalized using a Batch Normalization (BN) layer to stabilize training and improve gradient flow. The process of AGSC is as show in Equation 2:

(2) M1′=Conv3×3(M1), M2′=Conv3×3(M2)M1″=Conv1×1(GP(M1′))M12′=(M1″⊗M1′)M12=Sig(BN(Conv3×3(ReLU(M12′⊕M1′))))

where *M*_1_ from the encoder and *M*_2_ from the decoder are the input maps, *M*_12_ is the output feature maps.

AGSC employs an attention mechanism to recalibrate encoder features, amplifying pest-relevant information (e.g., edges, textures, body parts) while suppressing background clutter. This ensures discriminative features propagate through skip connections. Embedded at each level, AGSC progressively refines multi-scale features, preserving fine details for high-resolution segmentation. Compared to standard skip connections, this lightweight attention mechanism adaptively focuses on task-relevant regions, making it particularly effective for complex field scenes where pests occupy small areas. It enhances retention of minute features—such as antennae, legs, and body markings—significantly improving segmentation accuracy.

### 3.4 MSFF

To effectively integrate multi-scale features from different decoder stages and enhance the representation of small pests that are easily submerged in complex backgrounds, a MSFF is designed with hierarchical scale-aware attention, as illustrated in **Figure 2D**. The module takes the output features from four decoder stages, denoted as.

To effectively integrate multi-scale features from different decoder stages and enhance the representation of small pests that are easily submerged in complex backgrounds, a MSFF is designed with hierarchical scale-aware attention, as illustrated in **Figure 2D**. The module takes the output features from four decoder stages, denoted as *F*1​,*F*2​,*F*3​,*F*4​ and progressively fuses them through adjacent scale interactions. As shown in in **Figure 2D**, the infusion process follows a bottom-up hierarchical structure. Starting from the deepest layer, *F*4​ is first fused with *F*3​ through a scale-aware block. The fused result then interacts with *F*2​, and subsequently with *F*1​. This iterative process is described as show in Equation 3:

(3) $$\begin{array}{l} F_{3}^{\prime }=SA(F_{3},F_{4})\\ F_{2}^{\prime }=SA(F_{2},F_{3}^{\prime })\\ F_{1}^{\prime }=SA(F_{1},F_{2}^{\prime }) \end{array}$$

where $F_{i}(i=1,2,3,4)$ are features generated by the decoder and have been upsampled, *SA* denotes the scale-aware block with independent parameters for each fusion stage.

The detailed operation of each scale-aware block is illustrated in the lower part of **Figure 2D**. Given two adjacent scale features, taking $F_{3},F_{4}$ as an example, they are first concatenated along the channel dimension and passed through a convolutional layer to generate fusion weights. A Softmax activation is then applied to produce attention maps along the channel dimension. These attention maps are split to obtain channel-wise weighting coefficients for each input feature. The fused feature is computed as a weighted sum as follows:

(4) $$F_{3}^{\prime }=M_{3}⊙F_{3}+M_{4}⊙F_{4}$$

where *M*_3_ and *M*_4_​ are the corresponding attention maps generated by the Softmax layer, and ⊙ is element-wise multiplication.

In Equation 4, *F*_3_ and *F*_4_​ are concatenated and fed into convolution and Softmax layers, then the output is split along the channel dimension to obtain *M*_3_ and *M*_4_​.

This hierarchical fusion mechanism can achieve adaptive multi-scale feature refinement at each stage of the decoder. By gradually integrating features from deeper layers (rich in semantic information) with those from shallower layers (rich in spatial details), this module can ensure the effective preservation and enhancement of fine insect features (such as antennae, legs, and body markings), thereby significantly improving the accuracy of small target segmentation in complex environments.

### 3.5 Loss function

To optimize the proposed AGMSF-VMUNet for accurate CPD, a hybrid loss function combining cross-entropy loss and Dice loss is adopted to balance pixel-wise classification accuracy and region-wise detection consistency, defined as show in Equation 5:

(5) $$Loss=L_{\text{CE}}+\alpha L_{\text{DC}}$$

where *L_CE_* is cross-entropy loss, *L_DC_* is Dice loss, *α* is an adjustment coefficient, and the default value is 0.4.

*L_CE_* and *L_DC_* are defined as show in Equation 6:

(6) $$\begin{array}{l} L_{CE}=-\sum_{i}T_{i}\log(P_{i})\\ L_{DC}=1-2\sum_{i}T_{i}P_{i}/(\sum_{i}T_{i}+\sum_{i}P_{i}) \end{array}$$

where *T_i_* denotes the true label of the *i*th point in feature map *Z*, *P_i_* is the predicted probability.

This hybrid loss leverages the complementary strengths of both functions: cross-entropy ensures pixel-wise discrimination, while Dice loss enforces region-level completeness, together enabling precise segmentation of pests with varying sizes and fine details under complex field conditions.

### 3.6 Comparison with existing VM-UNet variants

To clarify the novelty of AGMSF-VMUNet, we compare it against three representative VM-UNet-based methods: VM-UNet (Ruan et al., 2024), Mamba-UNet (Wang et al., 2024), and LightM-UNet (Liao et al., 2024). Unlike VM-UNet, Mamba-UNet, and LightM-UNet, our AGMSF-VMUNet introduces three key innovations: MSVSS integrates four-scale dilated convolutions (1,3,5,7) for multi-scale feature extraction; AGSC replaces vanilla skip connections with spatial-channel attention to suppress background clutter; and MSFF progressively fuses features across four decoder stages using scale-aware attention. These task-specific designs—tailored for small targets, complex backgrounds, and multi-scale pest variation—distinguish our method from generic segmentation architectures.

## 4 Experiment on IP102 dataset

Experiments are conducted on the IP102 dataset to evaluate the performance of the proposed AGMSF-UNet against state-of-the-art models, including U-Net (Zhang et al., 2025), dilated multi-scale attention U-Net (DMSA-UNet) (Wang et al., 2024), SwinU-Net (Cao et al., 2023), VM-UNet (Ruan et al., 2024), and LightM-UNet (Liao et al., 2024), where U-Net and VM-UNet serve as baselines. Segmentation performance is quantified using the mean Intersection over Union (mIoU) and Dice Similarity Coefficient (DSC), calculated as show in Equation 7:

(7) $$mIoU=\frac{1}{N}\sum_{i=1}^{N}\frac{\left|Pred_{i}\;\cap \;GT_{i}\right|}{\left|Pred_{i}\cup GT_{i}\right|},\;DSC=\frac{1}{N}\sum_{i=1}^{N}\frac{2|Pred_{i}\;\cap \;GT_{i}|}{\left|Pred_{i}|+|GT_{i}\right|}$$

where *Pred_i_* and *GT* ​denote the predicted segmentation mask and ground truth for the *i*-th image, respectively, and |·| is the total number of pixels in the calculated set.

### 4.1 IP102 dataset

**Figure 3:**
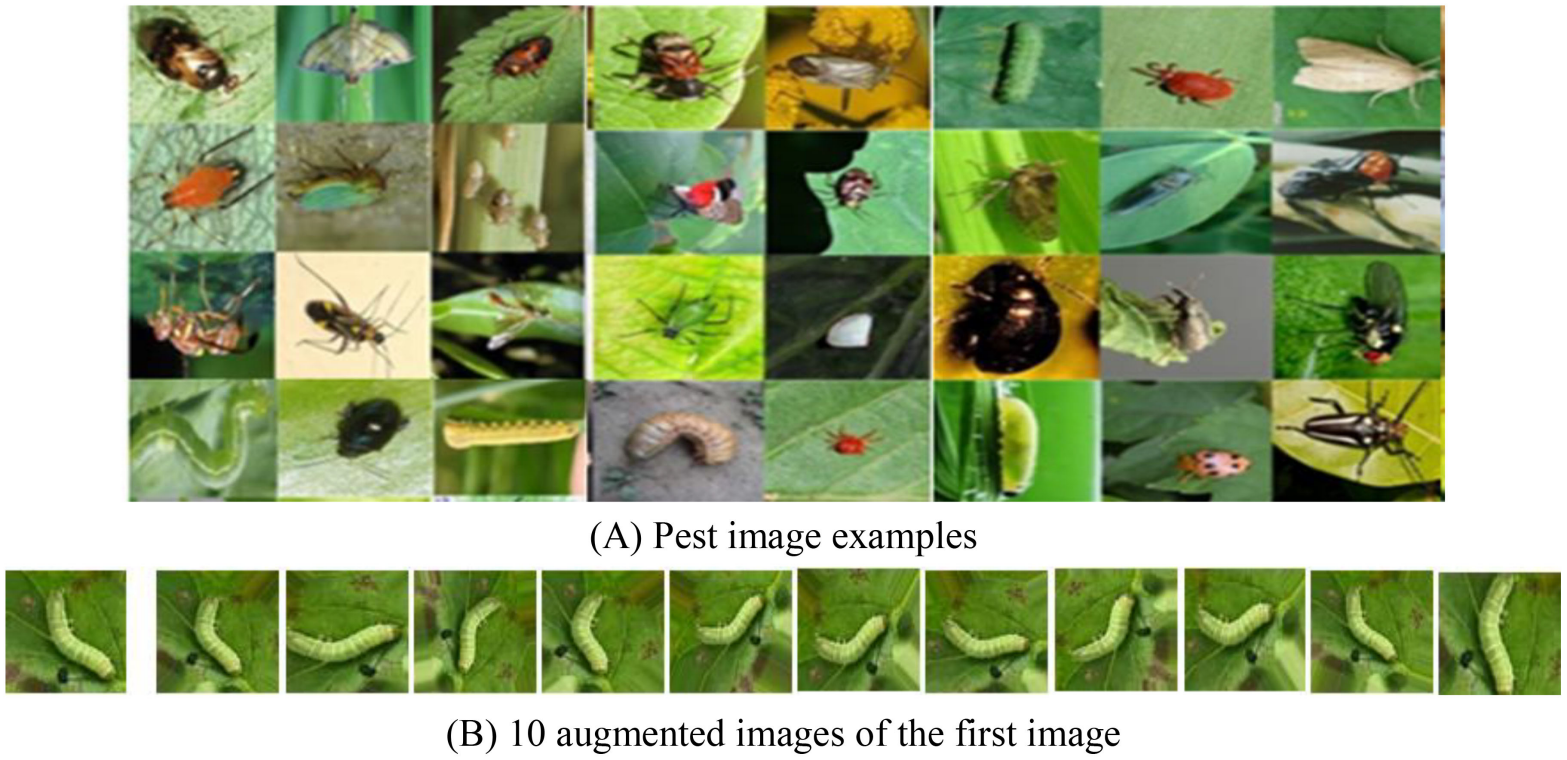
Examples of the pest images and corresponding augmented images. **(A)** Pest image examples. **(B)** 10 augmented images of the first image.

Experiments are conducted on the IP102 dataset, a large-scale benchmark for insect pest detection and identification (https://github.com/xpwu95/IP102) (Wu et al., 2019). The dataset contains over 75,000 images covering 102 pest categories across eight crops, including rice, corn, wheat, citrus, mango, sugar beet, alfalfa, and grapes. It exhibits significant class imbalance, reflecting real-world conditions. For instance, the smallest class contains 173 images, while the largest contains 1,115 images. Additionally, approximately 19,000 images are provided with bounding box annotations for pest detection tasks. Representative pest images are shown in **Figure 3A**, illustrating the dataset’s complexity, including varying scales, irregular backgrounds, and challenging field conditions.

**Data augmentation.** To address class imbalance and enhance detection capability, data augmentation techniques—including random translation, scaling, rotation, cropping, flipping, and mirroring—are applied to minority class subsets, increasing the number of training samples, as illustrated in **Figure 3B**.

Segmentation mask construction. Using IP102’s bounding boxes, we generated initial masks via GrabCut, then two experts manually refined them with LabelMe (correcting boundaries, annotating fine structures). A third expert resolved disagreements (κ=0.92). Quality control on 10% of masks achieved 96.7% consistency. The 480-hour effort produced segmentation masks for 102 pest categories across 75,000 images (Rother et al., 2004).

### 4.2 Experiment environment and parameter settings

All experiments are conducted under identical conditions to ensure fair comparison. The experimental environment consists of a Windows 10 operating system, an Intel Xeon E5–2667 v3 CPU @ 3.20 GHz, an NVIDIA Quadro M4000 GPU, with Python 3.8.8, PyTorch 1.10, and CUDA 11.3.

Hyperparameter selection. To ensure fair comparison, all models are trained using identical hyperparameters. The selection was based on a combination of prior work and grid search on a 10% validation split. The search ranges are: learning rate *lr* {1×10^-4^, 5×10^-4^, 1×10^-^³, 5×10^-^³}, batch size {8, 12, 15, 16}, momentum {0.3, 0.5, 0.7}, weight decay {1×10^-5^, 1×10^-4^, 1×10^-^³}, and Dice loss weight α {0.2, 0.3, 0.4, 0.5, 0.6}.

The selected values are: learning rate = 0.001 (following VM-UNet (Ruan et al., 2024) and LightM-UNet (Liao et al., 2024)), batch size = 15 (maximum feasible on our GPU), momentum = 0.5 (standard for SGD in segmentation tasks), weight decay = 0.0001 (grid search optimal), and α = 0.4 (grid search optimal). Total training iterations are set to 3,000, as validation metrics plateaued after approximately 2,500 iterations.

The experiment results present sensitivity analysis for the two most influential hyperparameters: learning rate and α. The results show that the selected values (*lr* = 0.001, α=0.4) achieve optimal or near-optimal mIoU, with performance degrading when deviating from these values.

**Figure 4:**
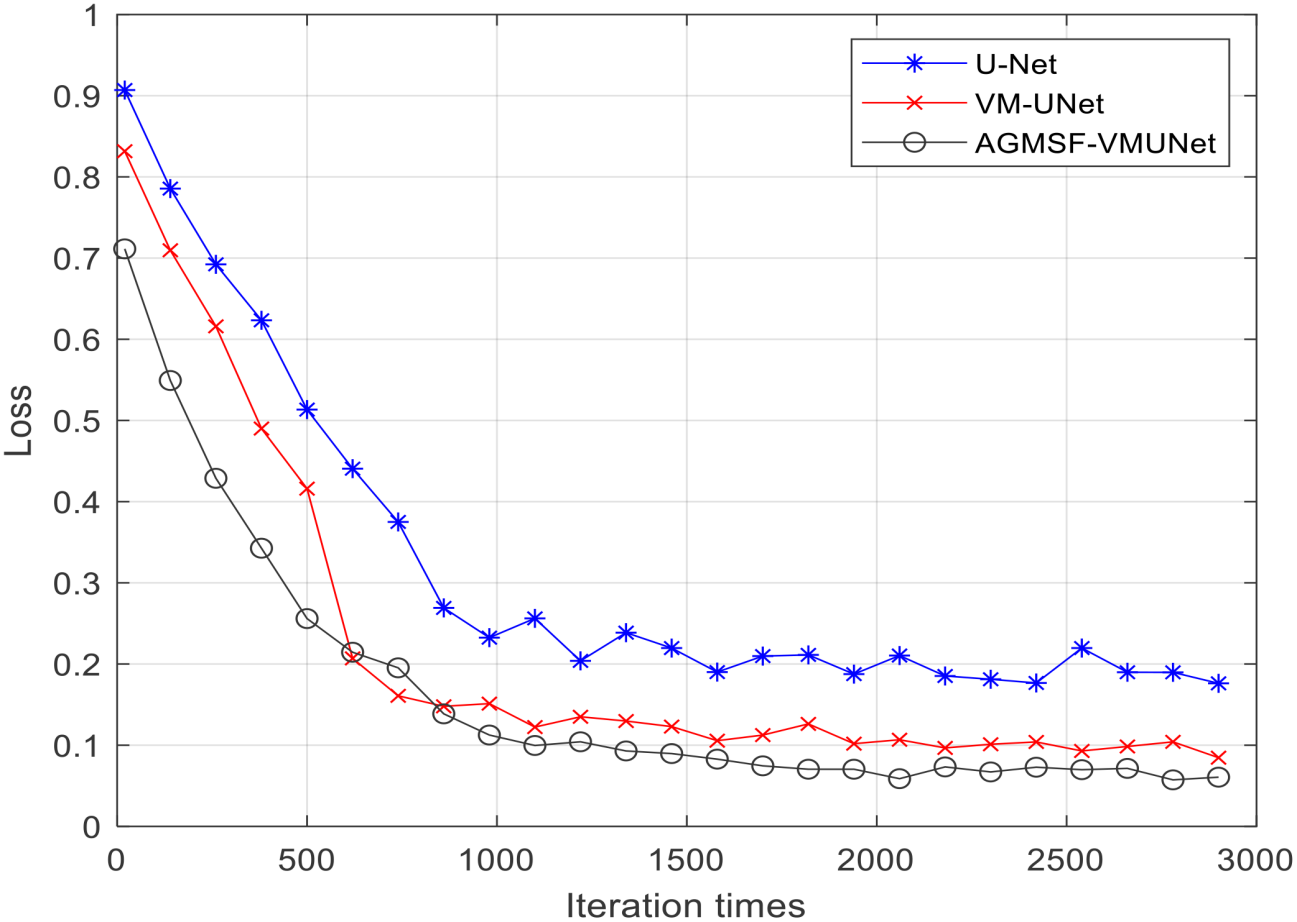
Training loss vs. iterations for U-Net, VM-UNet, and AGMSF-VMUNet.

All models are trained and fine-tuned using 5-fold cross-validation on the augmented IP102 dataset. **Figure 4** shows the training loss curves of U-Net, VM-UNet, and AGMSF-VMUNet across iterations. As observed in **Figure 4**, all three models converge stably after approximately 2,900 iterations, with AGMSF-VMUNet achieving the fastest convergence and the lowest final loss after 2,500 iterations. To ensure fair comparison, all models are trained for a fixed 3,000 iterations.

### 4.3 Visualization of detection results

**Figure 5:**
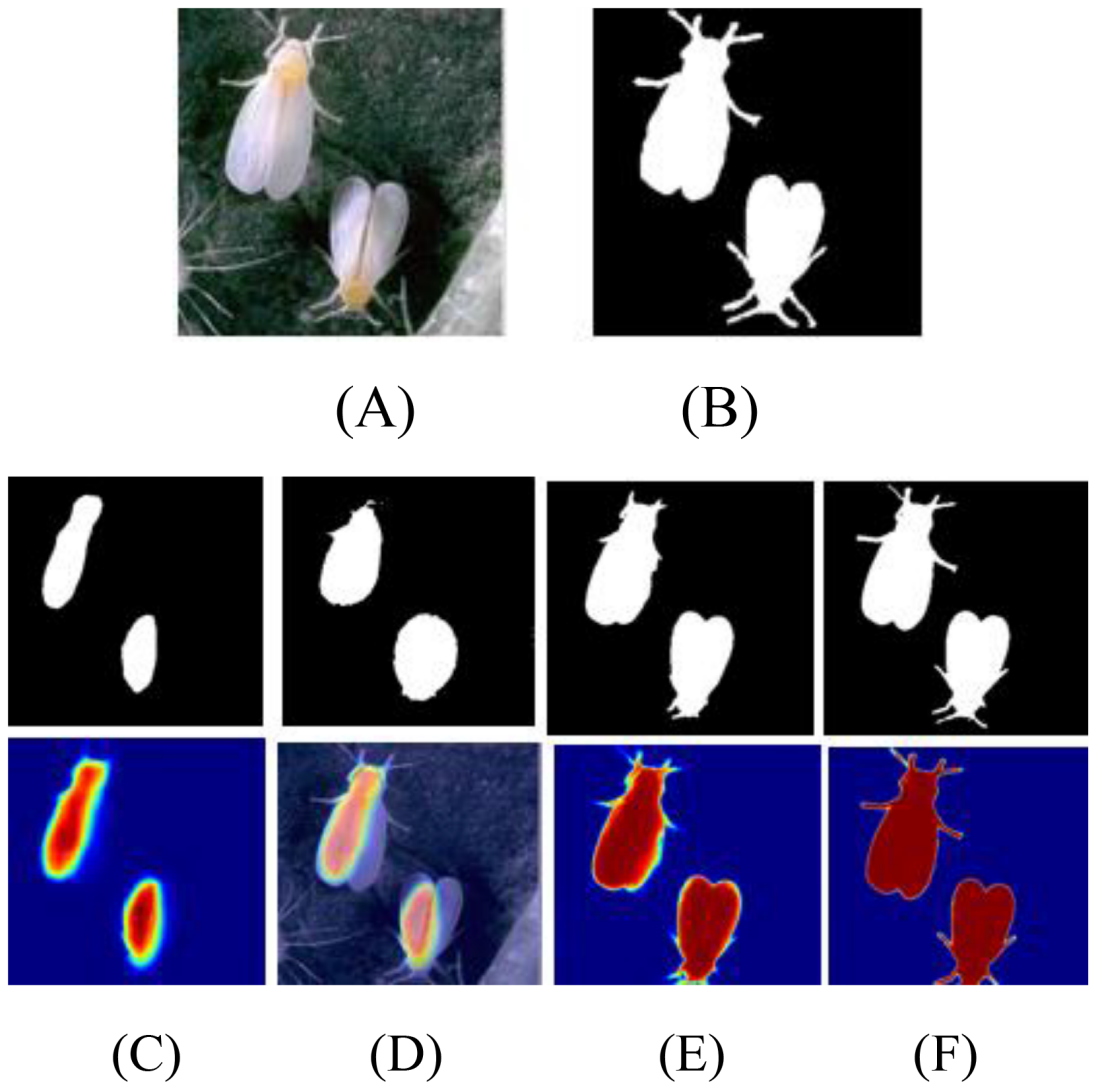
Detected pest images and heatmaps at different decoder stages where **(A)** Original image of crop pests, **(B)** Annotated images, **(C)** Stage 1, **(D)** Stage 2, **(E)** Stage 3, **(F)** Stage 4.

Feature heatmaps. **Figure 4** shows the segmented pests and heatmaps at different decoder stages (Stage 1 to Stage 4) by AGMSF-VMUNet. From **Figure 5**, it is seen that shallower layers (Stage 1–2) focus on low-level features such as edges, textures, and fine structures (e.g., antennae and body contours), while deeper layers (Stage 3–4) capture high-level semantic shape information. This hierarchical feature representation confirms that MSVSS effectively captures both local details and global context.

Experimental settings for fair comparison. For comparative evaluation, AGMSF-VMUNet is trained alongside U-Net, DMSA-UNet, SwinU-Net, VM-UNet, and LightM-UNet using 5-fold cross-validation, with the model checkpoint at the 3000th iteration selected for testing. All comparison models adopt identical settings: same data augmentation (random rotation ±15°, scaling 0.8–1.2, flipping, brightness adjustment), same optimizer (SGD, momentum 0.5), same training iterations (3,000 with early stopping), same input size (512×512), same loss function (Dice + CE, α=0.4), and same evaluation protocol (5-fold CV). The revised section explicitly states that all models were trained under identical conditions to ensure fair comparison. For classification-based models (e.g., SwinU-Net), we adapted the output head for segmentation while keeping all other settings identical. All models were trained under identical conditions to ensure fair comparison.

**Figure 6:**
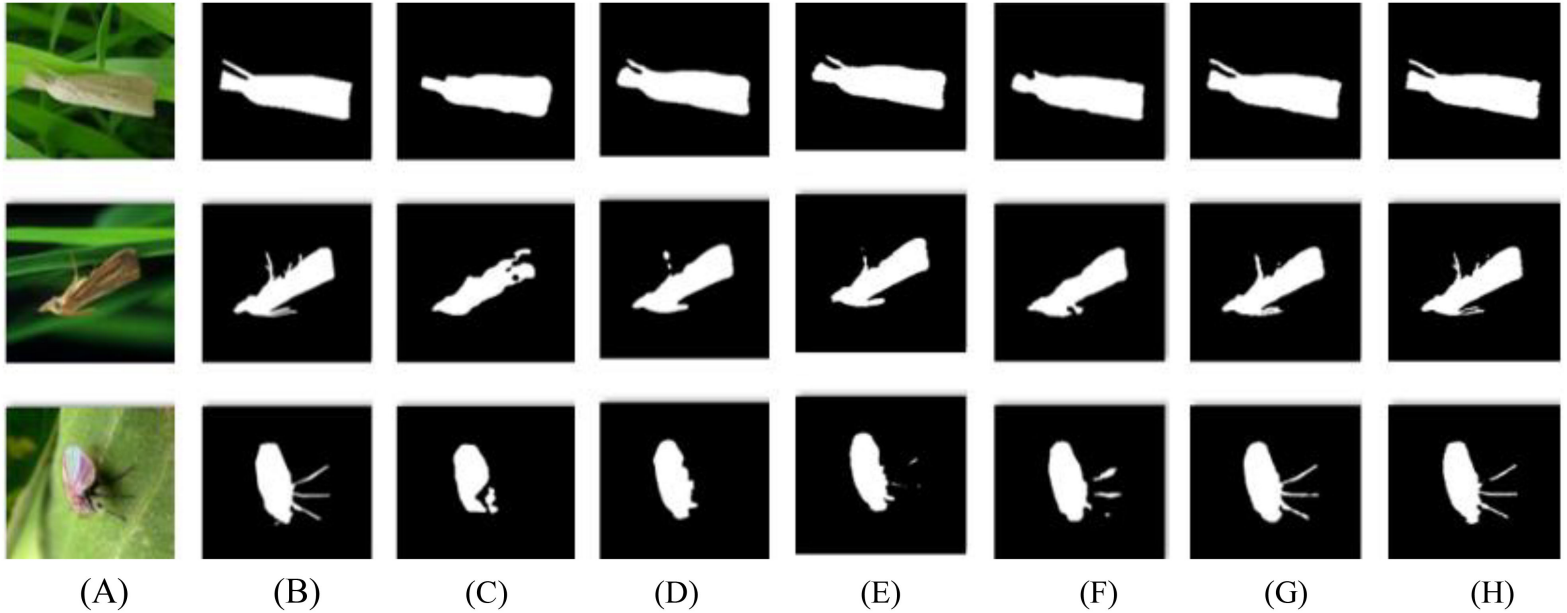
Detected pest images by six models, where **(A)** Original image of crop pests, **(B)** Annotated images, **(C)** U-Net, **(D)** DMSA-UNet, **(E)** SwinU-Net, **(F)** VM-UNet, **(G)** LightM-UNet, **(H)** AGMSF-VMUNet.

Segmentation results on simple images. **Figure 6** presents the visual segmentation results of these six models on representative simple crop pest images, where pests appear relatively large with clear shapes and uncomplicated backgrounds.

As shown in **Figure 6A**, each image presents a relatively simple scenario with large, clearly shaped pests and uncomplicated backgrounds. **Figure 6B** shows the corresponding annotated images. From **Figures 6C-H**, it can be observed that all six models successfully segment pests from these images. Among them, AGMSF-VMUNet delivers the best segmentation performance, accurately segmenting complete and clear antennae, legs, and head structures with well-defined edges. LightM-UNet achieves the second-best performance. The primary reason for this superior performance is that both models effectively capture key features of pests at different scales through their multi-scale representation mechanisms.

**Figure 7:**
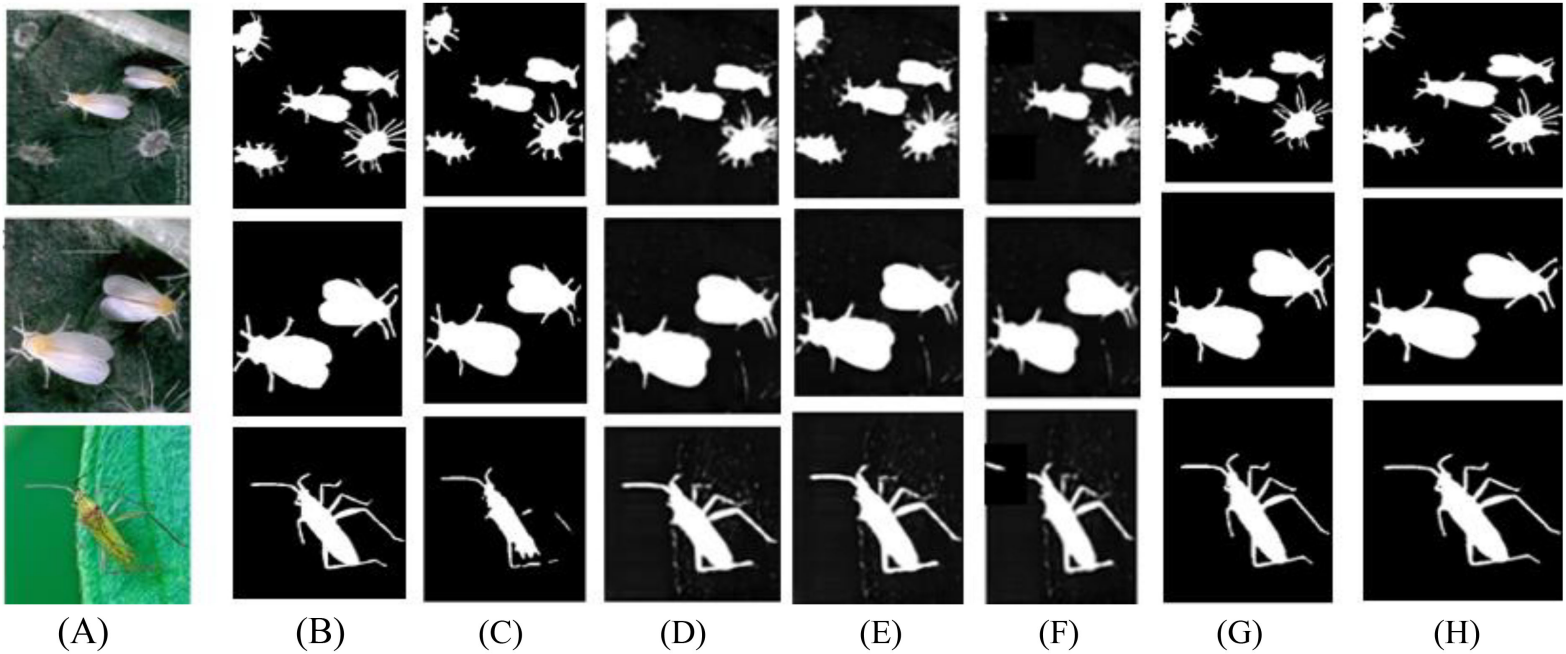
Detected complex pest images by six models, where **(A)** Original image of crop pests, **(B)** Annotated images, **(C)** U-Net, **(D)** DMSA-UNet, **(E)** SwinU-Net, **(F)** VM-UNet, **(G)** LightM-UNet, **(H)** AGMSF-VMUNet.

Segmentation results on complex images. To further validate the effectiveness and generalization of AGMSF-VMUNet, **Figure 7A** presents five complex pest images characterized by blurriness, small-scale and multi-scale pests, intricate backgrounds, low pest-background contrast, and dense populations of tiny pests in the last two images. Such challenging conditions make CPD particularly difficult for traditional methods. **Figure 7B** shows the corresponding annotated images. **Figures 7C–H** present the segmentation results obtained by the six compared models.

As observed in **Figures 5C–H**, AGMSF-UNet achieves the best segmentation performance, producing pests with relatively complete shapes and clearly visible structures, including complete antennae and the tiny legs of small pests. In contrast, U-Net and VM-UNet yield incomplete segmentations for some small pests, with noticeable loss of detailed features.

### 4.4 Quantitative segmentation results

To quantitatively verify the effectiveness of the proposed AGMSF-UNet model, comprehensive comparisons are conducted against five representative models: U-Net (Zhang et al., 2025), dilated multi-scale attention U-Net (DMSA-UNet) (Wang et al., 2024), SwinU-Net (Cao et al., 2023), VM-UNet (Ruan et al., 2024), and LightM-UNet (Liao et al., 2024). **Table 1** reports the Params (M), FLOPs (G), mean Intersection over Union (mIoU), Dice Similarity Coefficient (DSC), and training time for all models under identical experimental conditions.

**Table 1:** CPD results of six models.

Result model	Params (M)	FLOPs (G)	mIoU (%)	DSC (%)	Training time (h)
U-Net	31.02	55.8	63.26 ± 1.24	64.66 ± 1.18	11.28
DMSA-UNet	34.18	62.1	71.19 ± 0.92	73.32 ± 0.88	11.52
SwinU-Net	89.46	142.3	72.64 ± 0.85	75.22 ± 0.81	15.94
VM-UNet	45.23	82.3	75.09 ± 0.67	76.49 ± 0.64	6.52
LightM-UNet	12.76	24.1	75.23 ± 0.62	77.72 ± 0.59	6.59
AGMSF-VMUNet	68.42	108.6	77.22 ± 0.58	80.21 ± 0.55	7.37

AGMSF-VMUNet achieves the highest mIoU (77.22% ± 0.58%) and DSC (80.21% ± 0.55%), outperforming VM-UNet by 2.13% and 3.72% with a moderate increase in parameters (+23.19M) and FLOPs (+26.3G). Compared to LightM-UNet, it gains +1.99% mIoU and +2.49% DSC with higher computational cost. Against larger models like SwinU-Net (89.46M, 142.3 GFLOPs), AGMSF-VMUNet uses fewer resources yet achieves superior accuracy. The smallest standard deviations confirm its robustness. These results demonstrate a favorable trade-off between accuracy and efficiency.

### 4.5 Ablation experiments

To validate the effectiveness of each key component in the proposed AGMSF-VMUNet, comprehensive ablation studies are conducted on the IP102 dataset under identical experimental conditions. The baseline model is VM-UNet (Ruan et al., 2024), which employs standard VSS blocks in the encoder-decoder architecture with simple skip connections. Based on this baseline, we progressively integrate the proposed modules: MSVSS, AGSC and MFFM. The hybrid loss function is applied consistently across all variants. **Table 2** presents the quantitative results of different model variants.

**Table 2:** Ablation study results on IP102 dataset.

Variant no.	MSVSS	AGSC	MSFF	Hybrid loss	mIoU (%)	DSC (%)	Training time (h)
A (VM-UNet)	–	–	–	–	75.09	76.49	6.52
B	✓	–	–	–	76.28	77.83	6.78
C	–	✓	–	–	75.84	77.21	6.71
D	–	–	✓	–	76.12	77.58	6.89
E	–	–	–	✓	75.63	77.05	6.60
F	✓	✓	–	–	76.73	78.42	6.95
G	✓	✓	✓	–	77.05	79.38	7.18
H (AGMSF-VMUNet)	✓	✓	✓	✓	77.22	80.21	7.37

The ablation experiments demonstrate that each proposed module contributes positively to the overall performance of AGMSF- VMUNet:

MSVSS enhances multi-scale feature extraction, improving mIoU by 1.19% and DSC by 1.34% over the baseline. AGSC suppresses background interference via attention-guided skip connections, yielding 0.75% and 0.72% improvements. MFFM enables adaptive MSFF across decoder stages, boosting mIoU by 1.03% and DSC by 1.09%. Hybrid Loss addresses class imbalance through Dice loss, further improving mIoU by 0.54% and DSC by 0.56%. When combined, these modules exhibit strong synergistic effects. The full AGMSF-UNet achieves the best performance—77.22% mIoU and 80.21% DSC—representing substantial gains of 2.13% and 3.72% over the baseline VM-UNet, with only a modest increase in training time (+0.85 h). These results confirm the effectiveness of the proposed components and their collaborative contribution to accurate crop pest detection under complex field conditions.

### 4.6 Inference efficiency

To evaluate the practical deployability of AGMSF-VMUNet for real-world agricultural monitoring, we benchmarked inference performance on two representative hardware platforms. **Table 3** reports inference speed and hardware requirements for deployment.

**Table 3:** Inference efficiency on different platforms.

Platform	Model	Latency (ms)	FPS	GPU memory (GB)
GPU (Quadro M4000)	U-Net	18.2	54.9	1.8
	VM-UNet	24.5	40.8	2.4
	LightM-UNet	9.8	102.0	1.2
	**AGMSF-VMUNet**	31.7	31.5	3.1
Edge (Raspberry Pi 4B, INT8)	LightM-UNet	240	4.2	–
	**AGMSF-VMUNet**	312	3.2	–

The bold values indicate the model of AGMSF-VMUNet proposed in the paper.

The results in **Table 3** demonstrate that AGMSF-VMUNet can be deployed on edge devices with acceptable inference speed while maintaining high accuracy.

## 5 Experiment on AP162 dataset

**Figure 8:**
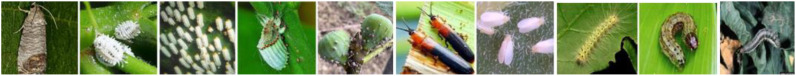
Original pest image examples.

To further validate generalization, additional experiments are conducted on the AP162 dataset (https://github.com/SCNYDX-KL/AP162). The dataset is publicly available and serves as an ideal benchmark for pest classification and segmentation tasks, containing 162 pest categories and 194,700 images. **Figure 8** shows some original pest image examples.

**Figure 9:**
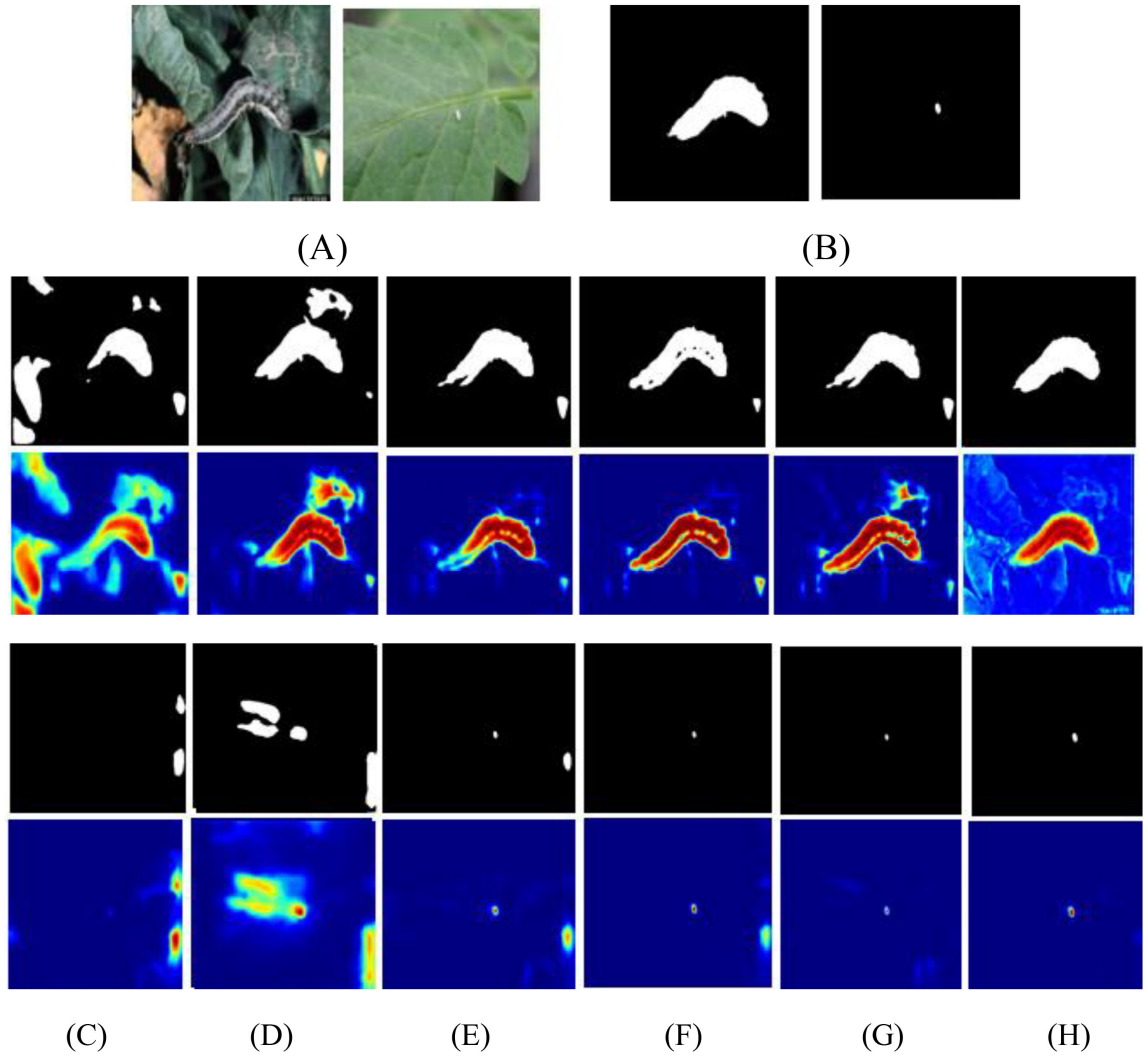
Qualitative comparison of pest detection on AP162 dataset. **(A)** Original images, **(B)** Ground truth masks, **(C)** U-Net, **(D)** DMSA-UNet, **(E)** SwinU-Net, **(F)** VM-UNet, **(G)** LightM-UNet, **(H)** AGMSF-VMUNet.

Following the identical experimental protocol described above for IP102, all models (U-Net, DMSA-UNet, SwinU-Net, VM-UNet, LightM-UNet, and AGMSF-VMUNet) are trained and evaluated on AP162 under the same hardware environment, hyperparameter settings (learning rate 0.001, batch size 15, momentum 0.5, weight decay 0.0001, 3,000 iterations), hybrid loss function (Dice + cross-entropy, α = 0.4), and 5-fold cross-validation strategy. Data augmentation techniques are also applied to minority classes to address class imbalance. **Figure 9** shows the detected pest images by six models.

From the segmentation results and heatmap visualizations in **Figure 9**, AGMSF-VMUNet demonstrates clear advantages over the compared models. U-Net and DMSA-UNet produce incomplete masks with blurred boundaries and severe target fragmentation. SwinU-Net and VM-UNet fail to separate distributed small pests, resulting in adhesion and missed detections. LightM-UNet achieves better performance but still loses fine details. In contrast, AGMSF-VMUNet generates complete, continuous pest contours with clear boundaries, preserving fine structures even for small and dense targets. This benefits from MSVSS and AGSC (enhancing pest-relevant information while suppressing background clutter).

## 6 Conclusion

To address the challenges of small scale, diverse morphology, and complex backgrounds in crop pest detection (CPD), an AGMSF-VMUNet model is constructed based on the VM-UNet framework. It is an attention-guided MSFF network for accurate crop pest detection in complex field environments. The model integrates three key components: Multi-Scale VSS (MSVSS) blocks for capturing both global context and local details with linear complexity, Attention Gate Skip Connections (AGSC) for dynamically enhancing pest-relevant features while suppressing background interference, and a progressive Multi-scale Feature Fusion (MSFF) for adaptively integrating features across decoder stages to preserve fine-grained details. Extensive experiments on the IP102 dataset demonstrate that AGMSF-UNet achieves state-of-the-art performance with 77.22% mIoU and 80.21% DSC, significantly outperforming existing CNN, Transformer, and SSM-based methods. Ablation studies confirm the synergistic contribution of each component, and visual analyses show robust segmentation under both simple and complex field conditions. Future work will focus on model lightweighting for edge deployment, domain adaptation for diverse agricultural environments, and larger-scale real-scenario datasets for practical validation.

## Data Availability

The original contributions presented in the study are included in the article/supplementary material. Further inquiries can be directed to the corresponding author.

## References

[B1] Abdullah H. Mohana N. Khan B. Ahmed S. Hossain M. Islam K. . (2023). Present and future scopes and challenges of plant pest and disease (P&D) monitoring: Remote sensing, image processing, and artificial intelligence perspectives. Remote Sens. Applications: Soc. Environ. 32, 100996. doi: 10.1016/j.rsase.2023.100996 38826717

[B2] Biradar N. Hosalli G. (2024). Segmentation and detection of crop pests using novel U-Net with hybrid deep learning mechanism. Pest Manag Sci. 80, 3795–3807. doi: 10.1002/ps.8083 38506377

[B3] Cao H. Wang Y. Chen J. Jiang D. Zhang X. Tian Q. . (2023). SwinU-Net: U-Net-like pure transformer for medical image segmentation. Lect Notes Comput. Sci. 13803. doi: 10.1007/978-3-031-25066-8_9 25273812

[B4] Domingues T. Brandão T. Ferreira J. (2022). Machine learning for detection and prediction of crop diseases and pests: A comprehensive survey. Agriculture 12, 1350. doi: 10.3390/agriculture12091350 30654563

[B5] Jin G. Zhang Q. Cheng Y. Xu M. Zhu Y. Yu D. . (2026). Enhancing feature discrimination with pseudo-labels for foundation model in segmentation of 3D medical images. Neural Networks 193, 107979. doi: 10.1016/j.neunet.2025.107979 40840292

[B6] Kiobia D. Mwitta C. Fue K. Schmidt J. M. Riley D. Rains G. . (2023). A review of successes and impeding challenges of IoT-based insect pest detection systems for estimating agroecosystem health and productivity of cotton. Sensors 23, 4127. doi: 10.3390/s23084127 37112469 PMC10146184

[B7] Liao W. Zhu Y. Wang X. Pan C. Wang Y. Ma L. (2024). LightM-UNet: Mamba assists in lightweight U-Net for medical image segmentation. arXiv, 2403.05246. doi: 10.48550/arXiv.2403.05246

[B8] Liu H. Zhan Y. Sun J. Mao Q. Wu T. (2025). A transformer-based model with feature compensation and local information enhancement for end-to-end pest detection. Comput. Electron. Agric. 231, 109920. doi: 10.1016/j.compag.2025.109920 38826717

[B9] Liu J. Yang H. Zhou H. Xi Y. Yu L. Yu Y. . (2024). Swin-UMamba: mamba-based U-net with imagenet-based pretraining. arXiv, 2402.03302. doi: 10.48550/arXiv.2402.03302

[B10] Mayya A. Alkayem N. (2025). Triple-stage crack detection in stone masonry using YOLO-ensemble, MobileNetV2U-Net, and spectral clustering. Autom Constr 172. doi: 10.1016/j.autcon.2025.106045 38826717

[B11] Nyawose T. Maswanganyi R. Khumalo P. (2025). A review on the detection of plant disease using machine learning and deep learning approaches. J. Imaging 11, 326. doi: 10.3390/jimaging11100326 41150002 PMC12565507

[B12] Pattnaik G. Parvathi K. (2022). Machine learning-based approaches for tomato pest classification. Telkomnika Telecommun Comput. Electron. Ctrl 20, 321–328. doi: 10.12928/telkomnika.v20i2.19740

[B13] Rahman M. Tutul A. Nath A. Laishram L. Jung S. K. Hammond T. (2024). Mamba in vision: A comprehensive survey of techniques and applications. arXiv, 2410.03105. doi: 10.48550/arXiv.2410.03105

[B14] Reddy D. Ramesh S. (2024). Identification of the pest detection using random forest algorithm and support vector machine with improved accuracy. AIP Conf. Proc. 3193, 020185. doi: 10.1063/5.0238448 42028584

[B15] Rother C. Kolmogorov V. Blake A. (2004). GrabCut": Interactive foreground extraction using iterated graph cuts. ACM Trans. Graph 23, 309–314. doi: 10.1145/3596711.3596774

[B16] Ruan J. Li J. Xiang S. (2024). VM-UNet: Vision mamba UNet for medical image segmentation. arXiv, 2402.02491. doi: 10.48550/arXiv.2402.02491

[B17] Saranya T. Deisy C. Sridevi S. (2024). Efficient agricultural pest classification using vision transformer with hybrid pooled multihead attention. Comput. Biol. Med. 177, 108584. doi: 10.1016/j.compbiomed.2024.108584 38788371

[B18] Shoaib M. Shah B. El-Sappagh S. Ali A. Ullah A. Alenezi F. . (2023). An advanced deep learning models-based plant disease detection: A review of recent research. Front. Plant Sci. 14, 1158933. doi: 10.3389/fpls.2023.1158933 37025141 PMC10070872

[B19] Wang Q. Wang C. Lai Z. Zhou Y. (2024). InsectMamba: Insect pest classification with state space model. arXiv, 2404.03611. doi: 10.48550/arXiv.2404.03611

[B20] Wang X. Zhang S. Zhang T. (2024). Crop insect pest detection based on dilated multi-scale attention U-Net. Plant Methods 20, 34. doi: 10.1186/s13007-024-01163-w 38409023 PMC10898010

[B21] Wang Z. Zheng J. Zhang Y. Cui G. Li L. (2024). Mamba-UNet: U-Net-like pure visual mamba for medical image segmentation. arXiv, 2402.05079. doi: 10.48550/arXiv.2402.05079

[B22] Wu X. Zhan C. Lai Y.-K. Cheng M.-M. Yang J. (2019). “ IP102: A large-scale benchmark dataset for insect pest recognition”, in: IEEE/CVF Conference on Computer Vision and Pattern Recognition (CVPR). (Piscataway, New Jersey (NJ): IEEE (Institute of Electrical and Electronics Engineers, IEEE Computer Society)), 8787–8796.

[B23] Xiang W. Du Z. Liu X. Lu Z. Yin Y. (2025). Research on the method of crop pest and disease recognition based on the improved YOLOv7-U-Net combined network. Appl. Sci. 15, 4864. doi: 10.3390/app15094864 30654563

[B24] Xu C. Yu C. Zhang S. Wang X. (2022). Multi-scale convolution-capsule network for crop insect pest recognition. Electronics 11, 248951630. doi: 10.3390/electronics11101630 30654563

[B25] Yang C. Zhao B. Mansurova M. Zhou T. Liu Q. Bao J. . (2025). AgriLiteNet: Lightweight multi-scale tomato pest and disease detection for agricultural robots. Horticulturae 11, 671. doi: 10.3390/horticulturae11060671 30654563

[B26] Yang X. Wang Q. Zhang K. Wei K. Lyu J. Chen L. (2025). MSV-Mamba: A multiscale vision mamba network for echocardiography segmentation. arXiv 11, 2501.07120. doi: 10.48550/arXiv.2501.07120

[B27] Yuan Y. Cheng Y. Pan B. Jin G. Yu D. Ye M. . (2025). A multi-modal attention fusion framework for road connectivity enhancement in remote sensing imagery. Mathematics 13, 3266. doi: 10.3390/math13203266 30654563

[B28] Zhang Q. Zhu Y. Cordeiro F. Chen Q. (2025). PSSCL: A progressive sample selection framework with contrastive loss designed for noisy labels. Pattern Recognit. 161, 111284. doi: 10.2139/ssrn.4903866

[B29] Zhang H. Wang R. Wang Z. Su W. (2025). DLCPD-25: A large-scale and diverse dataset for crop disease and pest recognition. Sensors 25, 7098. doi: 10.3390/s25227098 41305306 PMC12656478

[B30] Zhang S. Wang D. Yu C. (2023). Apple leaf disease recognition method based on Siamese dilated inception network with less training samples. Comput. Electron. Agric. 213, 108188. doi: 10.2139/ssrn.4329507

[B31] Zhang C. Zhang Y. Xu X. (2025). Dilated inception U-Net with attention for crop pest image segmentation in real-field environment. Smart Agric. Technol. 11, 100917. doi: 10.1016/j.atech.2025.100917 38826717

[B32] Zhang S. Zhang C. Yang C. Liu B. (2024). Editorial: Artificial intelligence and Internet of Things for smart agriculture. Front. Plant Sci. 15, 1494279. doi: 10.3389/fpls.2024.1494279 39416482 PMC11480055

